# Antidiabetic Effect of Artemether in Db/Db Mice Involves Regulation of AMPK and PI3K/Akt Pathways

**DOI:** 10.3389/fendo.2020.568864

**Published:** 2020-09-25

**Authors:** Xiaolin Bai, Ruixia Pei, Wen Lei, Meiyun Zhao, Jialin Zhang, Lu Tian, Junke Shang

**Affiliations:** Endocrinology Department, Xi'an Hospital of Traditional Chinese Medicine, Xi'an, China

**Keywords:** artemether, type 2 diabetes, Db/db mouse, AMPK, PI3K/Akt 3

## Abstract

The traditional Chinese medicine has long been used in the treatment of diabetes, one major disease threatening the public health. It has been reported that artemether exerts antidiabetic effects on type 2 diabetes in db/db mice, however the underlying mechanisms remain unknown. In the present study, we show that artemether regulates expression of related enzymes participating in the glucose and lipid metabolism in the liver of db/db mice, which could at least partly explain the improved glucose and lipid metabolism in artemether-treated mice. Additionally, artemether also regulates expression of glycogen synthesis related enzymes in the skeletal muscle of db/db mice, supporting its promotive role in glycogen synthesis. Mechanistically, artemether activates AMPK pathway as well as PI3K/Akt pathway in the liver and skeletal muscle of db/db mice, suggesting that these two signaling pathways are both involved in the antidiabetic effects of artemether on type 2 diabetes in db/db mice. In conclusion, our study connects the antidiabetic effects of artemether to the regulation of metabolic enzymes and signaling pathways, and also provides molecular basis for the potential application of artemether in treating type 2 diabetes.

## Introduction

Type 2 diabetes mellitus (T2DM) is a metabolic disease accounting for more than 90% of all diabetes cases (1). T2DM has become a global medical problem due to its high morbidity and mortality. The prevalence of T2DM, particularly in the developing countries, has been rapidly rising over the past three decades, and its number is estimated to reach 395 million by 2030, which represents 7.7% of the total population aged 20–79 years in the world (2). T2DM is characterized by increased blood glucose level, in which insulin resistance and insulin secretion failure are recognized as two main pathological changes that underlie its development and progression (3, 4). Currently, some Western medicine such as sulfonylurea, biguanides and thiazolidinediones have been widely employed to control T2DM symptoms, however, their efficacy is limited and some side effects also emerge, including increased appetite and weight (5, 6). Over two thousand years ago, the traditional Chinese medicine (TCM) were empirically used to treat diabetes-related symptoms, called “Xiaoke” disease (7). Nowadays, several TCM prescriptions have been proven to show clinical effectiveness in the treatment of T2DM, along with minimal side effects (8–10), suggesting a promising future for utilizing TCM in T2DM treatment. Artemisinin, derived from the Chinese herb *Artemisia annua*, is commonly used as a standard therapy for eliminating *Plasmodium falciparum malaria* (11). Aside from this, the role of artemisinin and derivatives in treating metabolic diseases has attracted increasing attention in recent years (12). For example, artemisinin and its derivatives exhibit certain anti-obesity and anti-fatty activities both *in vitro* and in animal models (13). Moreover, a previous study has shown that artemether has antidiabetic effects in db/db mice (14). However, the underlying mechanisms are still largely unclear.

In the present study, we report that artemether regulates the expression of glucose and lipid metabolism-associated enzymes in db/db mice. Subsequent evidence shows that artemether activates AMPK and PI3K/Akt pathways in the liver and skeletal muscle of db/db mice. Together, these data connect the antidiabetic effects of artemether in db/db mice to its role in the regulation of metabolic enzymes and signaling pathways.

## Materials and Methods

### Antibodies and Reagents

The primary antibodies against glucokinase, PEPCK, CPT1, and β-actin were purchased from Santa Cruz; The primary antibodies against PPARα, FAS, and GSK-3β were purchased from Abcam; The primary antibodies against GLUT4 and GYS were purchased from Novus Biologicals; The primary antibodies against p-ACC1, ACC1, p-AMPK, AMPK, p-PI3K, PI3K, p-AKT, and AKT were purchased from Cell Signaling. The secondary antibodies of goat-anti mouse and goat-anti-rabbit were purchased from Santa Cruz. Reagents of methylcellulose and artemether were purchased from Sigma-Aldrich.

### Animals

The male C57BL/KsJ-db/db mice and heterozygote C57BL/KsJ-db/+ littermates (6–8 weeks of age) were purchased from the Better Biotechnology Co., Ltd. (Nanjing, China). Mice were maintained in standard pathogen-free facilities (23 ± 1°C, 40–60% relative humidity, 12 h light-dark cycle) in the experimental animal center of our hospital. All mice were allowed free access to the normal chow diet and water during maintenance. The animal study was approved by the Animal Care and Use Committee and the Ethical Committee of Xi'an Hospital of Traditional Chinese Medicine.

### Artemether Treatment

After 2-weeks acclimatization, mice were randomly divided into four groups (*n* = 12) and treated as follows: normal control (NC, db/+, 1% methylcellulose, intragastric administration), diabetic control (DM Art-0, db/db, 1% methylcellulose, intragastric administration), artemether treated (DM Art-0.2, db/db, 200 mg/kg of artemether, intragastric administration) and artemether treated (DM Art-0.4, db/db, 400 mg/kg of artemether, intragastric administration). After 2 weeks of treatment and 8-h fasting, all mice were anesthetized and the whole blood was collected and plasma was isolated for biochemical analyses. The samples of skeletal muscle and liver were also excised immediately and stored at −80°C till use. All animal experiments were conducted in accordance with the guidelines approved by the Animal Care and Use Committee of Xi'an Hospital of Traditional Chinese Medicine.

### Western Blot Analysis

The total protein was extracted from the harvested skeletal muscle and liver using the One Step Animal Tissue Active Protein Extraction Kit (Sangon Biotech, C500006) according to the manufacturer's protocol. After protein quantification via the BCA method, protein samples were mixed with loading buffer and then denatured at 100°C for 5 min. The protein samples were separated by SDS-PAGE as previously described (15). In brief, equal amount of protein (30–50 μg) were loaded into 8%, 10% or 12% gel for electrophoresis and then transferred onto PVDF membrane (Millipore). Membrane was blocked with 5% BSA in TBST for 1 h at room temperature (RT), followed by overnight incubation with primary antibodies at 4°C and then 1-h incubation with corresponding secondary antibodies at RT. After repeated rinse with TBST, membrane was incubated with ECL Western Blotting Substrate (Pierce) to develop protein bands. Band densitometry was analyzed by ImageJ software.

### Plasma Lipid Measurement

The level of triglyceride (TG) and total cholesterol (TC) in mouse plasma was measured with the TG assay kit (Nanjing Jiancheng Bioengineering institute, A110-1) and TC assay kit (Nanjing Jiancheng Bioengineering institute, A111-1) according to the manufacturer's protocol. The absorbance of samples was recorded by the SpectraMax M5 Microplate Reader (Molecular Devices, Sunnyvale, CA, USA).

### Muscle Glycogen Measurement

After the sacrifice of mice, the skeletal muscle was immediately excised, washed with ice-cold saline, and stored at −80°C for glycogen measurement performed using the Glycogen Assay Kit (Sigma, MAK016) as described previously (16). Briefly, the frozen skeletal muscle (100 mg of each group) was thawed at RT and mixed with the alkali buffer and heated for 20 min with boiling water for glycogen extraction. Following the dilution with distilled water, the glycogen diluent was then mixed with the staining solution and heated for 5 min with boiling water. After cooling, the absorbance at 620 nm was determined using the SpectraMax M5 Microplate Reader (Molecular Devices, Sunnyvale, CA, USA). Glycogen content (mg/g of the skeletal muscle) was calculated using a glycogen standard curve. The final results are expressed as percentage of normal control.

### Statistics

All data are expressed as mean ± standard deviation (SD). The differences were calculated by one-way ANOVA using GraphPad Prism 6 software. *P* < 0.05 was considered to be statistically significant.

## Results

### Artemether Regulates Expression of Glucose Metabolic Enzymes in Db/Db Mouse Liver

**Figure 1:**
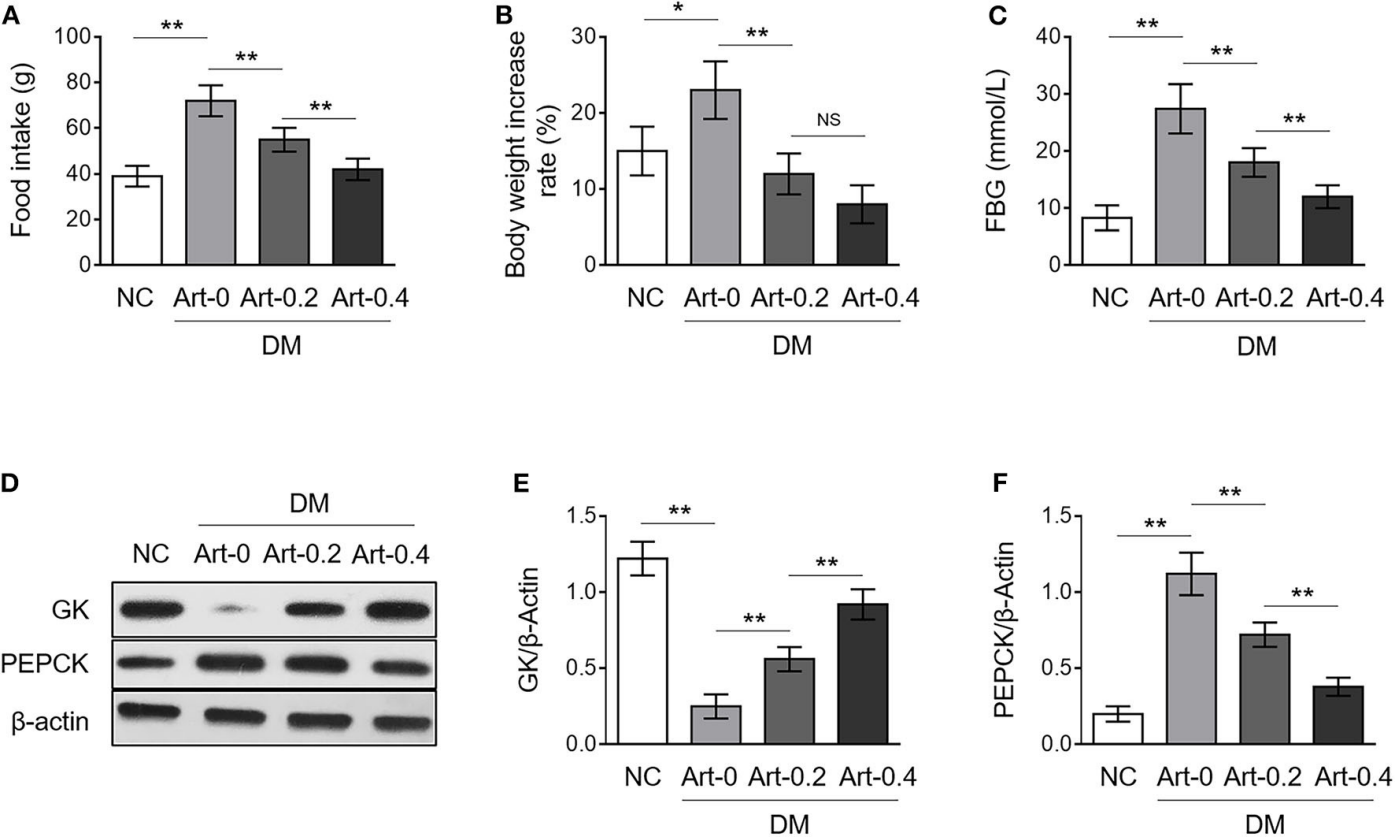
Artemether regulates expression of glucose metabolic enzymes in the livers of db/db mice. **(A–F)** Eight-week-old db/db mice were intragastrically administrated with 0 mg/kg (Art-0), 200 mg/kg (Art-0.2), or 400 mg/kg (Art-0.4) artemether every 2 days for consecutive 2 weeks. Meanwhile, 8-week-old littermate db/+ mice intragastrically administrated with 1% methylcellulose were used as controls (NC). Each group includes 12 mice. At the end of the experiment, the food intake **(A)**, body weight **(B)** and fasting blood glucose (FBG) **(C)** were measured. **(D)** The expression of glucokinase (GK) and phosphoenolpyruvate carboxykinase (PEPCK) in the liver samples was determined by Western blotting analysis. β-Actin was used as a loading control. The representative images from each group are shown. **(E,F)** The band intensity of GK **(E)** and PEPCK **(F)** from **(D)** was quantified by ImageJ and normalized to β-Actin. Each column represents the mean value of each group. Data are mean ± SD. Data were analyzed by one-way ANOVA test. **P < 0.01; *P < 0.05; NS, not significant.

Artemether decreases fasting blood glucose (FBG) level and improves glucose tolerance in db/db mice (diabetes mellitus, DM) (14). We wondered whether this effect of artemether is associated with altered glucose metabolism in the liver. To test this possibility, we checked the protein expression level of glucokinase (GK) and phosphoenolpyruvate carboxykinase (PEPCK), two critical rate-limiting enzymes individually controlling hepatic glycolysis and gluconeogenesis (17, 18), by Western blotting analysis using the liver samples obtained from db/+ mice in the negative control (NC) group and DM group administrated with or without artemether. These db/db mice naturally showed typical diabetic characteristics, like significant changes in food intake, body weight, and fasting blood glucose (FBG), which were dose-dependently reversed to some extent by artemether treatment (Figures 1A–C), as similarly reported by a previous study (14). More importantly, as shown in Figures 1D–F, compared with NC, GK expression was decreased and PEPCK expression was increased in the liver samples from DM mice (lane 1 vs. lane 2), suggesting a lower level of hepatic glycolysis but a higher level of gluconeogenesis in diabetic mice. However, artemether treatment significantly elevated GK expression and simultaneously reduced PEPCK expression in DM mice (Figures 1D–F, lane 2–4). These results suggest that artemether may decrease FBG level through increasing hepatic glycolysis and lowering gluconeogenesis via regulating the expression of GK and PEPCK in the livers of diabetic mice.

### Artemether Regulates Expression of Glycogen Synthesis Enzymes in Db/Db Mouse Skeletal Muscle

**Figure 2:**
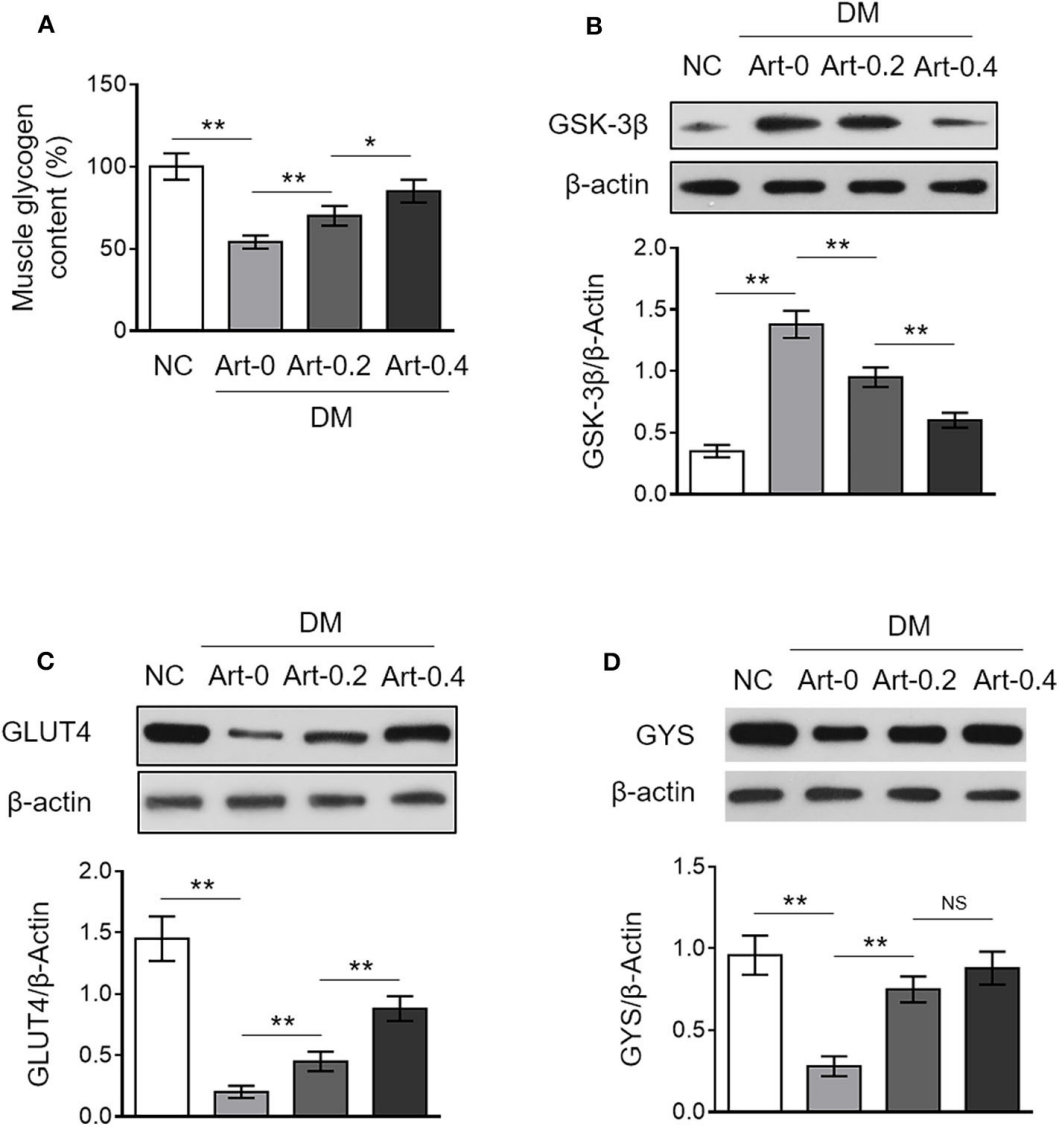
Artemether regulates expression of glycogen synthesis enzymes in the skeletal muscle of db/db mice. **(A–D)** Eight-week-old db/db mice were intragastrically administrated with 0 mg/kg (Art-0), 200 mg/kg (Art-0.2), or 400 mg/kg (Art-0.4) artemether every 2 days for consecutive 2 weeks. Meanwhile, 8-week-old littermate db/+ mice intragastrically administrated with 1% methylcellulose were used as controls (NC). Each group includes 12 mice. **(A)** The content of glycogen in the skeletal muscle was quantified. The results are expressed as relative to NC group (%). Each column represents the mean value of each group. Data are mean ± SD. Data were analyzed by one-way ANOVA test. **P < 0.01; *P < 0.05; NS, not significant. **(B–D)** The expression of GSK-3β **(B)**, GLUT4 **(C)**, and GYS **(D)** in the skeletal muscle was determined by Western blotting analysis. β-Actin was used as a loading control. The representative images (upper) and band intensity analysis (lower) are shown. Each column represents the mean value of each group. Data are mean ± SD. Data were analyzed by one-way ANOVA test. **P < 0.01; *P < 0.05; NS, not significant.

In addition to hepatic glycogen synthesis, glycogen synthesis in the skeletal muscle is also important for body glucose homeostasis (19). We found that the glycogen content in the skeletal muscle was much lower in DM mice compared with NC mice, and artemether treatment significantly increased it in DM mice (Figure 2A), illustrating that artemether recovers glycogen synthesis in the skeletal muscle of diabetic mice. The glycogen synthase kinase 3β (GSK3β), glucose transporter 4 (GLUT4) and glycogen synthase (GYS) play vital roles in controlling glucose uptake and glycogen synthesis in the skeletal muscle (20). Our results revealed that artemether markedly decreased GSK3β expression (Figure 2B), and meanwhile, increased expression of GLUT4 and GYS in DM mice (Figures 2C,D). Together, these findings point to that artemether may decrease FBG level through promoting glycogen synthesis in the skeletal muscle via regulating the expression of GSK3β, GLUT4, and GSY.

### Artemether Regulates Expression of Lipid Metabolic Enzymes in Db/Db Mouse Liver

**Figure 3:**
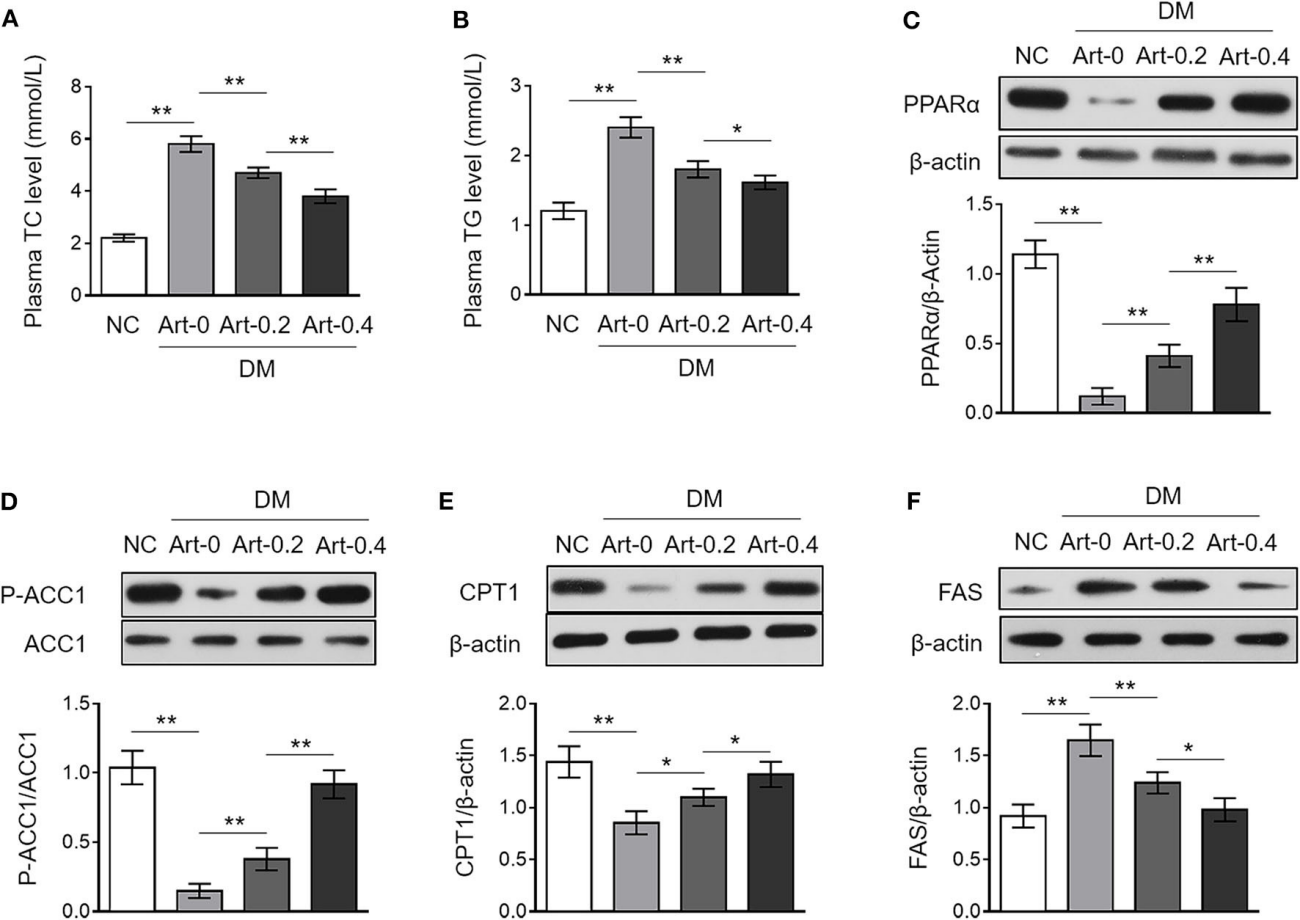
Artemether regulates expression of lipid metabolic enzymes in the livers of db/db mice. **(A–F)** Eight-week-old db/db mice were intragastrically administrated with 0 mg/kg (Art-0), 200 mg/kg (Art-0.2), or 400 mg/kg (Art-0.4) artemether every 2 days for consecutive 2 weeks. Meanwhile, 8-week-old littermate db/+ mice intragastrically administrated with 1% methylcellulose were used as controls (NC). Each group includes 12 mice. **(A,B)** The concentration of total cholesterol (TC) **(A)** and triglyceride (TG) **(B)** in the plasma was measured. **(C–F)** The expression of PPARα **(C)**, p-ACC1 **(D)**, CPT1 **(E)**, and FAS **(F)** in the liver was determined by Western blotting analysis. β-Actin or ACC1 was used as a loading control. The representative images (upper) and band intensity analysis (lower) are shown. Each column represents the mean value of each group. Data are mean ± SD. Data were analyzed by one-way ANOVA test. **P < 0.01; *P < 0.05.

Artemether has been shown to exhibit anti-obesity function (14, 21), but whether it affects the level of plasma lipid is not investigated yet. We show here that both the levels of triglyceride (TG) and total cholesterol (TC) in the plasma were increased in DM mice compared with those of NC group, whereas, artemether treatment significantly reduced them in DM mice (Figures 3A,B), which indicates a beneficial role of artemether in preventing the increase of plasma lipid level in diabetic mice. The enzymes, including peroxisome proliferator-activated receptor alpha (PPARα), acetyl-CoA carboxylase 1 (ACC1) and carnitine palmitoyl transferase 1 (CPT1), are important regulators of hepatic lipid metabolism (22, 23). Notably, we found that DM mice had lower expression levels of hepatic PPARα, phosphorylated ACC1, and CPT1 than those of NC mice, however, which were all recovered to marked extent when DM mice were treated with artemether (Figures 3C–E). PPARα retards *de novo* fatty acid synthesis via inhibiting the fatty acid synthase (FAS) that catalyzes fatty acid synthesis from acetyl-CoA and malonyl-CoA (24). Interestingly, we noticed that the upregulated expression of hepatic FAS in DM, as compared with NC mice, was significantly decreased by artemether, even declining to the level of NC mice when relatively high dosage of artemether (400 mg/kg) was administrated (Figure 3F). Collectively, these observations imply that artemether reduces plasma lipid level in diabetic mice, which may be related to the increased expression of hepatic PPARα, ACC1 and CPT1, as well as decreased hepatic FAS expression.

### Artemether Activates AMPK Pathway in the Liver and Skeletal Muscle From Db/Db Mice

**Figure 4:**
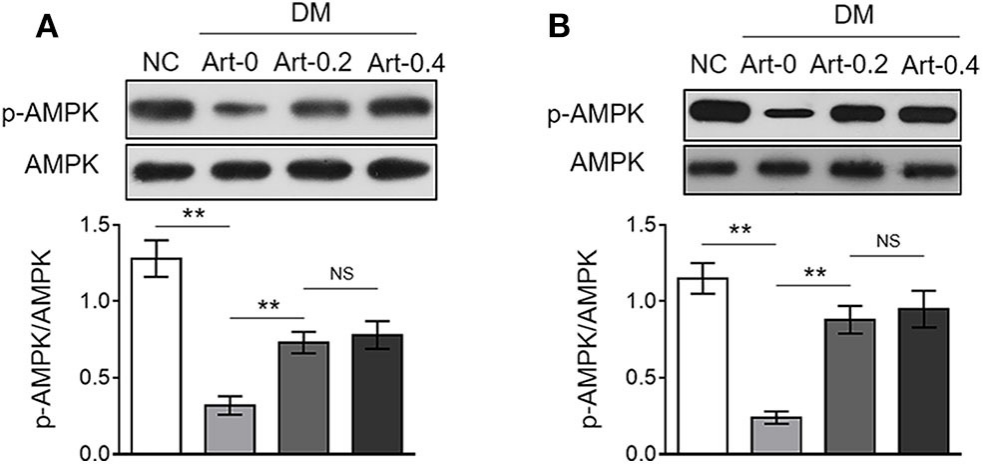
Artemether activates AMPK pathway in the liver and skeletal muscle of db/db mice. **(A,B)** Eight-week-old db/db mice were intragastrically administrated with 0 mg/kg (Art-0), 200 mg/kg (Art-0.2), or 400 mg/kg (Art-0.4) artemether every 2 days for consecutive 2 weeks. Meanwhile, 8-week-old littermate db/+ mice intragastrically administrated with 1% methylcellulose were used as controls (NC). Each group includes 12 mice. The expression of p-AMPK and AMPK in the liver **(A)** and skeletal muscle **(B)** was determined by Western blotting analysis. The representative images (upper) and ratio of p-AMPK/AMPK (lower) are shown. Each column represents the mean value of each group. Data are mean ± SD. Data were analyzed by one-way ANOVA test. **P < 0.01; NS, not significant.

The AMP-activated protein kinase (AMPK) pathway is a central cellular energy sensor that regulates glucose and lipid metabolism (25). To understand the molecular mechanism of the anti-diabetic function of artemether, we checked its possible effect on AMPK pathway in the liver and skeletal muscle from NC and DM mice. The results showed that AMPK activity was lower in both the liver (Figure 4A) and skeletal muscle (Figure 4B) from DM mice than that of NC mice, as evidenced by the decreased level of phosphorylated AMPK (p-AMPK). But, oppositely, artemether treatment significantly reversed AMPK activity in DM mice (Figures 4A,B). Hence, these data suggest that artemether may regulate glucose and lipid metabolism in the liver and skeletal muscle by modulating AMPK pathway in diabetic mice.

### Artemether Activates PI3K/Akt Pathway in the Liver and Skeletal Muscle From Db/Db Mice

**Figure 5:**
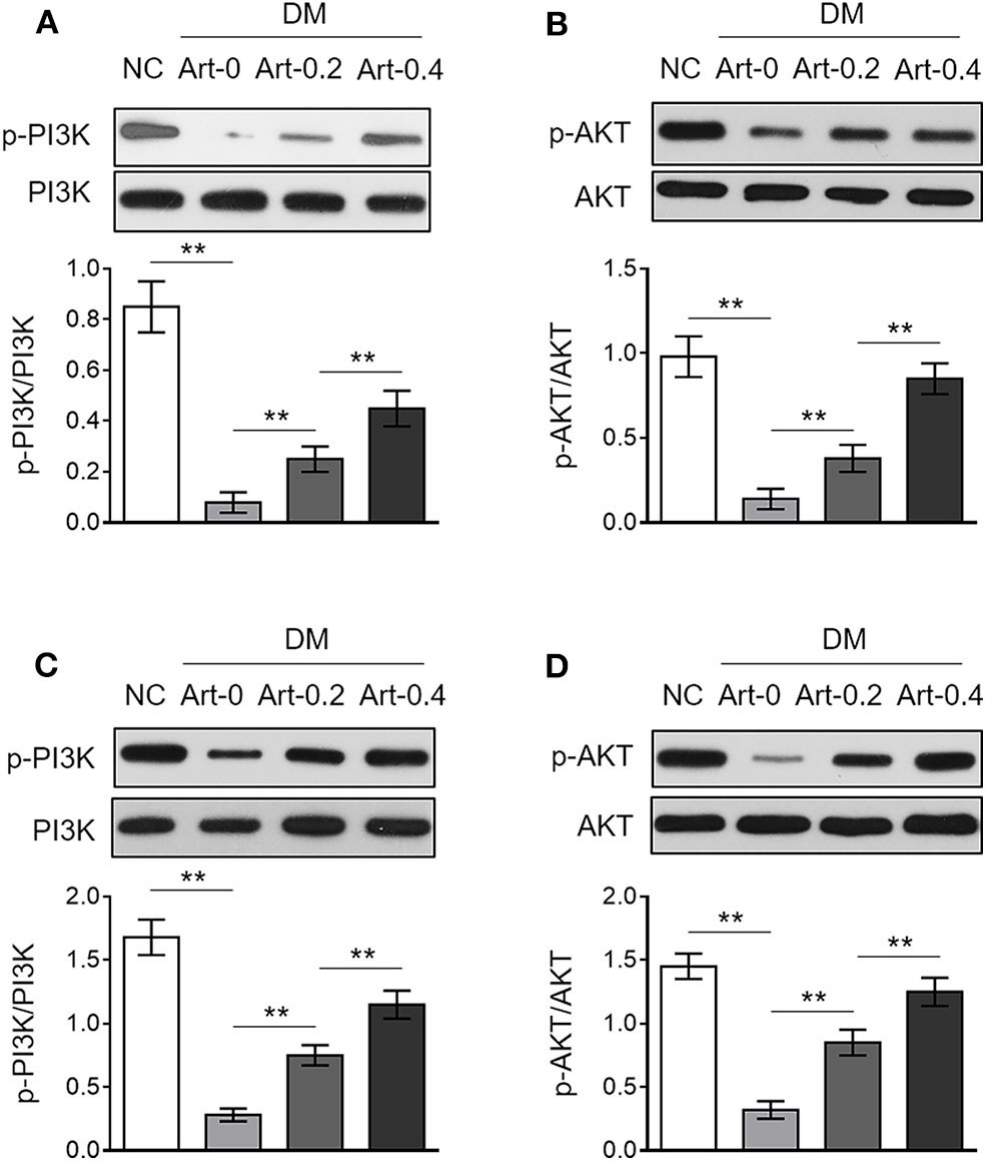
Artemether activates PI3K/Akt pathway in the liver and skeletal muscle of db/db mice. **(A–D)** Eight-week-old db/db mice were intragastrically administrated with 0 mg/kg (Art-0), 200 mg/kg (Art-0.2), or 400 mg/kg (Art-0.4) artemether every 2 days for consecutive 2 weeks. Meanwhile, 8-week-old littermate db/+ mice intragastrically administrated with 1% methylcellulose were used as controls (NC). Each group includes 12 mice. The expression of p-PI3K, PI3K, p-Akt, and Akt in the liver **(A,B)** and skeletal muscle **(C,D)** was determined by Western blotting analysis. The representative images (upper) and ratio of p-PI3K/PI3K and p-Akt/Akt (lower) are shown. Each column represents the mean value of each group. Data are mean ± SD. Data were analyzed by one-way ANOVA test. **P < 0.01.

**Figure 6:**
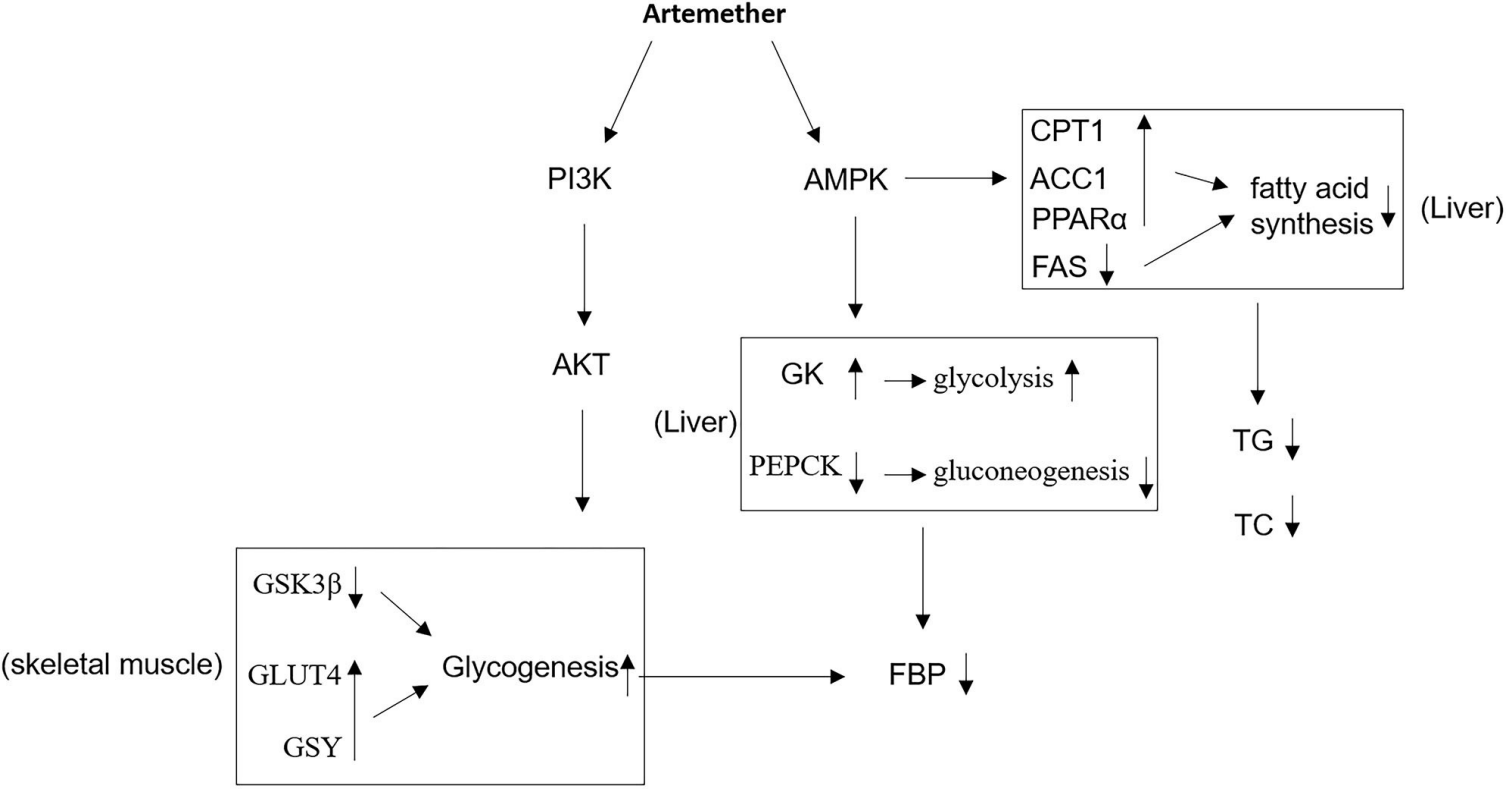
Proposed mechanisms of antidiabetic activities of artemether. In db/db mice, artemether activates AMPK and PI3K/Akt pathways, through which it affects the expression of metabolic enzymes that regulate glucose and lipid metabolism, contributing to the improved glucose homeostasis and reduced obesity. GK, glucokinase; PEPCK, phosphoenolpyruvate carboxykinase; FBG, fasting blood glucose; GSY, glycogen synthase; TG, triglyceride; TC, total cholesterol; FAS, fatty acid synthase.

The phosphatidylinositol 3-kinase/protein kinase B (PI3K/Akt) pathway downstream of insulin receptor is responsible for glucose uptake and glycogenesis (26). We therefore asked whether this pathway may also be related to diabetic function of artemether. To test this, we detected the expression of phosphorylated PI3K and Akt in the liver and skeletal muscle from NC and DM mice by Western blotting analysis. As a result, in contrast to NC mice, the levels of phosphorylated PI3K (Figure 5A) and Akt (Figure 5B) were decreased in the liver of DM mice, which were, to large extent, elevated by artemether treatment, indicating that the PI3K/Akt pathway is activated by artemether. Moreover, similar results were obtained when the activity of PI3K/Akt pathway in the skeletal muscle was compared between NC mice and DM mice treated with or without artemether (Figures 5C,D). Taken together, the above lines of evidence suggest that the regulated glucose (Figures 1, 2) and lipid (Figure 3) metabolic enzymes and the activated metabolic AMPK (Figure 4) and PI3K/Akt (Figure 5) pathways in the liver and skeletal muscle may contribute to the antidiabetic function of artemether in diabetic mice (Figure 6).

## Discussion

Artemether, a derivative of artemisinin, has been previously demonstrated to possess antidiabetic activities in db/db mice, such as improving glucose homeostasis and insulin resistance, preventing obesity and reducing the severity of fatty liver, and protecting pancreatic beta cells (14). Based on these observations, we proposed that artemether might serve as a promising and effective medicine for the treatment of T2DM (14). However, the underlying molecular mechanisms by which artemether exerts these antidiabetic activities are unclear, which could dismay the rational application of artemether against T2DM in the future. The present study was designed to explore how artemether improves the glucose and lipid metabolism in db/db mice, focusing on examining the potential regulatory role of artemether in the protein expression of enzymes associated with glucose and lipid metabolism and metabolic signaling pathways, including AMPK and PI3K/Akt, in mouse liver and skeletal muscle.

Based on the evidence, we conclude that the antidiabetic activities of artemether may at least partly be attributed to two major mechanisms. Firstly, the regulation of the expression of glucose and lipid metabolic enzymes in the liver and skeletal muscle, including GK and PEPCK, two critical enzymes controlling the hepatic glycolysis and gluconeogenesis; GSK3β, GLUT4, and GSY, three enzymes associated with glycogen synthesis in skeletal muscle; CPT1, ACC1, PPARα, and FAS, four enzymes regulating lipid metabolism in the liver. Secondly, the activation of two metabolic signaling pathways, including AMPK pathway and PI3K/Akt pathway in the liver and skeletal muscle. Overall, it seems that artemether exhibits a wide-spectrum of effects on glucose and lipid metabolism to reduce blood glucose level and fatty acid synthesis for fulfilling its antidiabetic function.

AMPK is an energy sensor that coordinates cellular metabolism by regulating the status of several downstream target effectors commonly involved in glucose and lipid metabolism (27). In addition, the elevated production of glucose in the liver plays a fundamental role for developing hyperglycemia, one typical symptom of T2DM. Serving as a negative regulator, the activation of AMPK decreases hepatic glucose production by means of stimulating glycolytic enzymes and simultaneously suppressing gluconeogenic enzymes in the liver (28). In this study, we found that artemether treatment activated AMPK pathway in both the liver and skeletal muscle of db/db mice, as shown by elevated expression of p-AMPK. Moreover, coinciding with AMPK activation, artemether treatment elevated GK expression and reduced PEPCK expression in DM mice, which are two key enzymes determining hepatic glycolysis and gluconeogenesis in the liver. Therefore, these results not only relate the hypoglycemic activity of artemether in db/db mice to its function in regulating the expression of GK and PEPCK, but also suggest that the activated AMPK pathway at least in part contributes to artemether-regulated glycolysis and gluconeogenesis in the liver.

In addition to GK and PEPCK, AMPK pathway also regulates the lipid metabolic enzymes in the liver, including CPT1, ACC1, PPARα, and FAS (29). We discovered that artemether treatment significantly reduced the levels of both TG and TC in DM mice, and keeping in line with this result, the expression of p-ACC, PPARα, and CPT-1 was upregulated and FAS expression was downregulated by artemether treatment. This anti-obesity activity of artemether is consistent with findings as previously reported by other groups (14, 21). The data provided in this study connect the anti-obesity activity of artemether to the altered expression of AMPK pathway downstream targets, which are involved in lipid metabolism in the liver.

PI3K/Akt pathway is also an important signal transduction that regulates glucose metabolism, and its activation mediates the transduction of insulin receptor signaling by further regulating the downstream target proteins, including GSK3β, GLUT4, and GSY (30). Among the three targets, GSK3β negatively regulates glycogen synthesis in the skeletal muscle by promoting the phosphorylation of GYS and decreasing its enzymatic activity (20). GLUT4 is pivotal factor for skeletal muscle glucose uptake (31). Our results showed that artemether increased glycogen content, and accordingly, decreased GSK3β expression and increased expression of GLUT4 and GSY in the skeletal muscle of db/db mice, implying that artemether may promote glycogen synthesis through increasing glucose uptake via GLUT4, and meanwhile, accelerating catalytic reaction via inhibiting GSK3β and activating GYS. Furthermore, we found that artemether activated PI3K/Akt pathway in db/db mice, thus associating the artemether-elevated glycogen synthesis with the expression change of GSK3β, GLUT4 and GSY, which are under the regulation of PI3K/Akt pathway.

In summary, our study reveals the antidiabetic activities of artemether in db/db mice, including decreasing blood glucose level, increasing glycogen synthesis, and reducing plasma lipid level. Our evidence further relates these antidiabetic activities to its stimulating effects on AMPK and PI3K/Akt pathways, whereby artemether is able to affect the expression of their downstream targets that participate in modulating glucose and lipid metabolism. Therefore, our study may provide some molecular basis for explaining how artemether improves glucose homeostasis and reduces obesity in db/db mice, which would benefit the potential application of artemether in T2DM treatment in the future.

## Data Availability Statement

All datasets presented in this study are included in the article/supplementary material.

## Ethics Statement

The animal study was reviewed and approved by Xi'an Hospital of Traditional Chinese Medicine.

## Author Contributions

JS conceived and designed the present study. XB and RP performed the experiments and analyzed the data. WL, MZ, JZ, and LT substantially contributed to drafting the manuscript. All authors read and approved the final manuscript.

## Conflict of Interest

The authors declare that the research was conducted in the absence of any commercial or financial relationships that could be construed as a potential conflict of interest.
